# Гинекологическая эндокринология и репродуктивная медицина с рождения до старости: в фокусе Институт репродуктивной медицины ГНЦ РФ ФГБУ НМИЦ эндокринологии

**DOI:** 10.14341/probl13534

**Published:** 2024-11-01

**Authors:** Р. К. Михеев, Е. Н. Андреева, О. Р. Григорян, Е. В. Шереметьева, Ю. С. Абсатарова, Ж. А. Ужегова

**Affiliations:** Национальный медицинский исследовательский центр эндокринологии; Национальный медицинский исследовательский центр эндокринологии; Российский университет медицины; Национальный медицинский исследовательский центр эндокринологии; Национальный медицинский исследовательский центр эндокринологии; Национальный медицинский исследовательский центр эндокринологии; Национальный медицинский исследовательский центр эндокринологии

**Keywords:** гинекология, эндокринология, онтогенез, рождение, менопауза, персонализация

## Abstract

Гинекологическая эндокринология (эндокринная гинекология) — уникальное интегративное направление репродуктивной медицины, сформировавшееся в последние 50 лет благодаря прорывным открытиям в области гинекологии, эндокринологии и терапии. Согласно действующему законодательству (приказ МЗ РФ от 07.10.2015 г. №700н) специальность «врач-эндокринолог-гинеколог» в классификаторе Минздрава России отсутствует, в то время как служба эндокринной гинекологии испытывает дефицит кадров из-за сложившейся необходимости подготовки врачей по 2 программам ординатуры: эндокринологии и акушерству и гинекологии. На сегодняшний день 80% обращений за гинекологической помощью обусловлены нарушениями эндокринного генеза, остальные 20% (инфекции, передающиеся половым путем, орфанные заболевания) — неэндокринного характера. Целью данной статьи является освещение современного состояния, актуальности развития службы эндокринной гинекологии в ГНЦ РФ ФГБУ НМИЦ эндокринологии в рамках оказания персонализированной помощи на всех этапах жизни женщины.

Статья посвящается 100-летию основания ГНЦ РФ ФГБУ НМИЦ эндокринологии Минздрава России.

## Abstract (en)

Gynecological endocrinology (also known as endocrine gynecology) is the unique integrative area of reproductive medicine. It was established circa 50 years ago due to breakthrough discoveries in the gynecology, endocrinology and internal medicine. According to Russian legislation there is no such specialization as endocrinologist-gynecologist but there is a lack of appropriate doctors due to complicated residenture post-diploma system in Russian national healthcare. Nowadays 80% gynecological disorders are related to endocrinopathies in opposite to 20% caused by sexually transmitted infections and orphan diseases. The aim of the present article is to present modern condition of female healthcare and actuality of endocrine gynecology service development in the Endocrinology Research Centre (Moscow, Russia).

The article is dedicated to the 100th anniversary of the National Research Centre for Endocrinology of the Russian Ministry of Health.

## ВВЕДЕНИЕ

Согласно научной концепции о физиологии функциональных систем, выдвинутой в XX в. академиком АН СССР П.К. Анохиным (1889–1974), женская репродуктивная система представляет собой многоуровневую иерархическую систему, направленную на достижение полезного результата — обеспечение менструальной, детородной и органопротективной функций. На протяжении всей жизни женщины, от рождения до глубокой старости, репродуктивная система неизбежно подвергается значительным функциональным нагрузкам, чреватым развитием различных вариантов коморбидности. Аккумуляция стресс-обусловленных сбоев гипоталамо-гипофизарно-яичниковой оси — с одной стороны, и заметное расширение клинико-лабораторных возможностей XXI в. — с другой, обеспечили в последние годы глобальный рост гинекологической заболеваемости. Задача по решению данной проблемы возложена на службу гинекологической эндокринологии (эндокринной гинекологии), первые попытки по организации которой были выполнены еще в 1930–1940-е гг. благодаря пионерским исследованиям д.м.н. Е.И. Кватера (1894–1972) [1]. Исторический прорыв в вопросах разработки мер профилактики, диагностики и лечения гормонально детерминированных нарушений репродуктивной системы состоялся благодаря организации в 1960 г. в стенах НИИЭиХГ (ныне — ГНЦ РФ ФГБУ НМИЦ эндокринологии Минздрава России) группы эндокринной гинекологии, заслуженно добившейся в наши дни статуса флагманского подразделения по своему профилю в масштабах Российской Федерации. Основополагающий вклад в формирование данного подразделения был внесен руководителями группы (ныне — отделения): д.м.н. И.В. Голубевой (1921–1986 гг.) и д.м.н. А.А. Пищулиным (1946–2005 гг.) [2]. В настоящий момент в рамках Института репродуктивной медицины (директор — д.м.н., проф. Е.Н. Андреева) ГНЦ РФ ФГБУ НМИЦ эндокринологии Минздрава России (директор — член-корр. РАН, д.м.н., проф. Н.Г. Мокрышева, президент — акад. РАН, д.м.н., проф. И.И. Дедов), в отделении эндокринной гинекологии, ежегодно получают помощь более 10 000 пациенток с гинекологической патологией эндокринного генеза.

## ЖЕНСКАЯ РЕПРОДУКТИВНАЯ СИСТЕМА В ПЕРИОДЕ ДЕТСТВА

В отличие от репродуктивной системы женщин фертильного периода, представленной пятью звеньями (ЦНС, вентро- и дорсомедиальные аркуатные ядра гипоталамуса, аденогипофиз, яичники, органы-мишени), для новорожденных девочек ввиду транзиторного влияния материнских (плацентарных) эстрогенов, супрессии по механизму отрицательной обратной выработки гипофизарных гонадотропинов и гонадотропин-рилизинг-гормон (ГнРГ) связи характерно сохранение активности только 3 звеньев [3]. Патогномоничным признаком функциональной активности репродуктивной системы у новорожденных девочек в течение первых 10 суток жизни вследствие остаточной активности плацентарных эстрогенов является т.н. синдром отмены материнских эстрогенов. К основным клиническим проявлениям данного состояния относятся нагрубание молочных желез [4], мажущие/капельные кровяные выделения из половых путей («микроменструации») [5]. В норме данное «кризовое» состояние самостоятельно купируется по мере клиренса материнских эстрогенов на 11-е сутки жизни и не требует медицинского вмешательства. До 7–9 лет репродуктивная система девочек находится в состоянии низкой функциональной активности, пока не наступает цепная реакция в составе активации кисспептин-вырабатывающих KNDy-нейронов аркуатного ядра гипоталамуса, последующего пульсационного высвобождения (в фолликулярной фазе — 1 раз в 60–90 мин, в лютеиновой фазе — каждые 2–4 часа) ГнРГ, а также частотозависимой выработки ФСГ и ЛГ аденогипофизом [6].

Нозологии, связанные с нарушениями репродуктивной системы в допубертатном периоде, находятся в зоне ответственности акушеров-гинекологов, эндокринологов и хирургов педиатрического профиля.

Амбулаторно-поликлиническая и стационарная помощь пациенткам детского и подросткового возраста успешно оказывается в ГНЦ РФ ФГБУ НМИЦ эндокринологии Минздрава России за счет коллаборации сотрудников Института детской эндокринологии (директор — д.м.н., проф. О.Б. Безлепкина) и Института репродуктивной медицины (директор — д.м.н., проф. Е.Н. Андреева).

## ЖЕНСКАЯ РЕПРОДУКТИВНАЯ СИСТЕМА В ПЕРИОДЕ ПОЛОВОГО СОЗРЕВАНИЯ

Половое созревание (пубертатный период; от лат. pubertas — половая зрелость) у девочек — это генетически программируемый и строго поэтапно структурированный физиологический процесс соматического и репродуктивного становления во втором десятилетии жизни с длительностью около 10 лет. Именно в этом периоде закладываются психофизиологические механизмы готовности к продолжению рода, происходит адаптация социальной активности к предстоящим циклическим гормональным изменениям. Происходит переход индивидуума женского пола из зоны ответственности педиатрической службы в зону ответственности акушера-гинеколога женской консультации, родильного дома, отделения эндокринной гинекологии, а в случае диагностируемого бесплодия — репродуктолога. Принято различать в данном процессе следующие этапы: препубертат (с 7–9 до 10–11 лет), I фаза (с 10–11 до 12–14 лет), II фаза (с 12–14 лет до 17–19 лет).

## Препубертат

Инициация пубертата осуществляется в первую очередь за счет постепенного повышения выработки в сетчатом слое надпочечников андрогенов, в частности ДГЭА и ДГЭА-С (адренархе). Выработка ДГЭА и ДГЭА-С (в комплексе с СТГ и ИФР-1) способствует удлинению трубчатых костей в процессе ростового скачка, достигая взрослых значений уже к 12 и 15 годам соответственно. Активизируется внегонадный синтез эстрогенов: андрогены (андростендион и тестостерон) начинают подвергаться в жировой ткани конверсии в эстрогены (эстрон и эстрадиол) за счет активности ароматазы (изоформы цитохрома Р-450), что способствует феминизации фигуры за счет расширения тазовых костей.

Примерно через 2–3 года после свершившегося адренархе инициируется импульсная секреция ГнРГ гипоталамусом — гонадархе, что приводит к активизации гипоталамо-гипофизарно-гонадной оси: в аденогипофизе запускается импульсная секреция гонадотропинов (ЛГ и ФСГ) в сочетании с СТГ, за счет синтеза рецепторов к гонадотропинам, на овариальных фолликулах обеспечивается синтез эстрогенов для органов-мишеней [7].

## I фаза

Пограничной временной точкой между препубертатом и I фазой пубертата является телархе (от др.-греч. θηλή — «сосок» и ἀρχή — «начало») — начало эстроген-ассоциированного увеличения молочных желез. Определяемое физикально увеличение молочных желез позволяет клиницисту констатировать факт свершившегося начала эстрогенизации организма девочки.

Следующим значимым событием является последовательное формирование характерного третичного волосяного покрова по женскому типу: в области лобка — в виде перевернутого треугольника основанием вверх (пубархе; от лат. pubis — «лобок»), а также в области подмышечных впадин (аксиллархе; от лат. axillaris — подмышечный). Для оценки динамики изменений волосяного покрова у пациенток принято использовать шкалу Маршалла-Таннера [8].

## II фаза

О начале II фазы пубертата сигнализирует наступление менархе (от др.-греч. μήν «месяц» + ἀρχή «начало») — первой менструации, уточнение возраста которой является необходимым пунктом при оформлении медицинской документации вне зависимости от профиля оказания помощи. Первая менструация — сигнал состоявшегося начала подготовки организма девочки к деторождению. Во II фазу пубертата происходит становление цирхорального ритма выработки ГнРГ в гипоталамусе, что способствует: цикличности секреции гонадотропинов в аденогипофизе, цикличности выработки эстрогенов и прогестерона в яичниках и впоследствии — установлению регулярного менструального цикла, либо сразу, либо в течение следующих 6–12 месяцев после менархе.

Зачастую менструации девочек во II фазу пубертата являются ановуляторными (у ~80%), нерегулярными и затянувшимися (до 7 суток и более).

У большинства девочек менструации начинаются в 12–14 лет, однако в генеральной совокупности данный показатель вариабелен ввиду наследственных, конституциональных факторов, коморбидности и питания. Принято считать, что условиями наступления менархе является масса тела 47–48 кг, а также доля жировой ткани 20–22% по данным биоимпедансометрии. Необходимо уточнить о негативном влиянии избытка жировой массы тела на репродуктивное здоровье в будущем: по данным исследования Deng P et al. (2023 г.) (n=118 291), объем висцеральной жировой ткани способствует ускорению возраста наступления менархе (β=-0,33; 95% ДИ -0,49; -0,16) и одновременно повышает риск развития преэклампсии в будущем при наступлении беременности (ОР 1,65, 95% ДИ 1,23–2,20) [9].

«Периферическим эффектом» менархе является эстроген-ассоциированное окостенение ростковых зон и торможение скорости линейного роста девочек; в дальнейшем, к 18 годам, рост девушки достигается инерционно, за счет увеличения длины позвоночного столба (1–3 см). За счет становления обратной связи эстрогены способствуют формированию пятиуровневой системы нейроэндокринной регуляции, характерной для женщин зрелого репродуктивного возраста.

Консультативно-диагностическая помощь женщинам репродуктивного вопроса осуществляется в ГНЦ РФ ФГБУ НМИЦ эндокринологии Минздрава России благодаря взаимодействию 3 подразделений: Отделения эндокринной гинекологии (заведущая отделением — д.м.н., профессор Е.Н. Андреева), Отделения андрологии и урологии (заведующий отделением — к.м.н. С.Н. Волков) и Отделения вспомогательных репродуктивных технологий (заведующая отделением — д.м.н. И.И. Витязева).

## ПЕРИ- И ПОСТМЕНОПАУЗАЛЬНЫЙ ПЕРИОД

Репродуктивный период сменяется этапом менопаузального перехода, за которым следуют менопауза и в итоге постменопауза. Менопаузальный переход — естественный этап утраты репродуктивной функции женщины и формирующейся полигландулярной недостаточности в исходе снижения синтеза прогестерона, эстрогенов и андрогенов. Падение уровня прогестерона клинически выражается формированием недостаточности лютеиновой фазы с последующим сокращением частоты овуляторных циклов, вплоть до их полного прекращения. Снижение уровня эстрогенов нередко приводит к манифестации симптомов климактерического синдрома: приливы жара и потливости, колебания эмоционального фона, сухость и зуд половых органов, болезненность при половых контактах (диспареуния), снижение либидо. Первоначально формирующаяся на фоне угасания функции яичников относительная гиперандрогения впоследствии сменяется дефицитом общего и свободного тестостерона. В результате, ввиду недостаточности половых гормонов, по механизму обратной связи формируется лабораторная картина гипергонадотропного гипогонадизма.

Пребывание в состоянии менопаузального перехода чревато структурными и метаболическими сдвигами, повышающими риск возраст-ассоциированных заболеваний: ишемической болезни сердца (ИБС), цереброваскулярной болезни (ЦВБ), сахарного диабета 2 типа, остеопороза и т.д. [10].

## ЗАДАЧИ ЭНДОКРИННОЙ ГИНЕКОЛОГИИ

По состоянию на 20-30-е гг. XXI в. задачи специалистов гинекологов-эндокринологов состоят в следующем.

Репродуктивные изменения, а именно снижение содержания половых гормонов, играют, с одной стороны, ведущую роль в процессах старения, а с другой — являются единственными на сегодняшний день возрастными изменениями, на которые можно эффективно воздействовать. Это обуславливает необходимость разработки мероприятий по охране репродуктивного здоровья старшей возрастной группы, которые приведут не только к повышению качества жизни и сохранению работоспособности, активного долголетия, но и значительно снизят расходы, направленные на лечение последствий возрастного гормонального дефицита (остеопороз, ожирение, сердечно-сосудистые заболевания).

Наиболее негативными тенденциями характеризуется репродуктивное и половое здоровье населения сельской местности, где возрастные гормональные изменения наступают на 10 лет раньше, что ведет к раннему старению и ранней инвалидизации. Антивозрастная помощь в сельских районах полностью отсутствует, что в еще большей степени обостряет ситуацию и ведет к миграции населения в города, где они могут рассчитывать на помощь и лечение. Необходимо уточнить, что все хронические соматические заболевания у женщин потенциально могут ускорять возрастное снижение секреции эстрогенов в среднем на 5–7 лет.

Авторы настоящей статьи считают, что эффективными юридическими мерами ликвидации острого дефицита кадров отечественной службы эндокринной гинекологии могли бы стать возвращение программы постдипломной подготовки в рамках интернатуры, усовершенствование системы первичной переподготовки врачей-эндокринологов и врачей-акушеров-гинекологов [11]. В настоящее время на базе ГНЦ ФГБУ «НИМЦ эндокринологии» проведено восемь ежегодных Международных конференций по репродуктивному здоровью женщин и мужчин, привлекших внимание к проблемам репродуктивного здоровья населения России тысячи специалистов (акушеров, гинекологов, эндокринологов, урологов, терапевтов, кардиологов, геронтологов, андрологов и врачей других специальностей) из разных уголков нашей страны и мира.

Профилактическая направленность работы гинеколога-эндокринолога, использование высоких технологий диагностики и лечения позволит не только сохранить репродуктивную функцию, здоровье, трудоспособность и улучшить демографическую ситуацию в стране, но и снизить ежегодные затраты по лечению и социальной помощи пациентам с эндокринопатиями при нарушениях репродуктивной функции.

Залогом успешной реализации работы службы эндокринной гинекологии является внедрение единых стандартов этапного обследования и лечения больных с эндокринными нарушениями репродуктивной функции во все возрастные периоды, с использованием новейших технологий, разрабатываемых в научных и лечебных подразделениях, а также персонифицированной медицины.

## ЗАКЛЮЧЕНИЕ

На сегодняшний день гинекологическая эндокринология (эндокринная гинекология) представляет собой одно из важнейших, бурно развивающихся направлений современной медицины. Кажущиеся современному человеку привычными методы оценки уровней половых гормонов, фармакологических проб, использование методов контрацепции и менопаузальной гормональной терапии дают возможность одновременно управлять детородной функцией, обеспечивать эмансипацию, снижать риск инвалидизации. Все вышеперечисленные результаты являются наглядным пособием хорошо организованной лечебно-диагностической работы и научно-образовательной деятельности в стенах ГНЦ РФ ФГБУ «НМИЦ эндокринологии» Минздрава России. Трепетное соблюдение принципов преемственности, коллегиальности и научного подхода способствует достижению цели сохранения и улучшения репродуктивного здоровья населения нашей страны. Институт репродуктивной медицины ГНЦ ФГБУ «НИМЦ эндокринологии» Минздрава России продолжает свою работу, вступая в новый век эндокринной гинекологии.

## ДОПОЛНИТЕЛЬНАЯ ИНФОРМАЦИЯ

Источники финансирования. Работа выполнена по инициативе авторов без привлечения финансирования.

Конфликт интересов. Авторы декларируют отсутствие явных и потенциальных конфликтов интересов, связанных с содержанием настоящей статьи

Участие авторов. Все авторы одобрили финальную версию статьи перед публикацией, выразили согласие нести ответственность за все аспекты работы, подразумевающую надлежащее изучение и решение вопросов, связанных с точностью или добросовестностью любой части работы.
